# Sphingosine 1-phosphate signaling in bone remodeling: multifaceted roles and therapeutic potential

**DOI:** 10.1080/14728222.2017.1332180

**Published:** 2017-06-07

**Authors:** Anastasia Meshcheryakova, Diana Mechtcheriakova, Peter Pietschmann

**Affiliations:** ^a^ Department of Pathophysiology and Allergy Research, Center for Pathophysiology, Infectiology and Immunology, Medical University of Vienna, Vienna, Austria

**Keywords:** Bone biology, bone diseases, coupling factor, osteoclast – osteoblast crosstalk, osteoporosis, osteotropic therapies, sphingosine 1-phosphate, sphingosine 1-phosphate receptor antagonist/agonist, sphingolipid-related checkpoints

## Abstract

**Introduction**: Sphingolipids belong to a complex class of lipid molecules that are crucially involved in the regulation of important biological processes including proliferation, migration and apoptosis. Given the significant progress made in understanding the sphingolipid pathobiology of several diseases, sphingolipid-related checkpoints emerge as attractive targets. Recent data indicate the multifaceted contribution of the sphingolipid machinery to osteoclast – osteoblast crosstalk, representing one of the pivotal interactions underlying bone homeostasis. Imbalances in the interplay of osteoblasts and osteoclasts might lead to bone-related diseases such as osteoporosis, rheumatoid arthritis, and bone metastases.

**Areas covered**: We summarize and analyze the progress made in bone research in the context of the current knowledge of sphingolipid-related mechanisms regulating bone remodeling. Particular emphasis was given to bioactive sphingosine 1-phosphate (S1P) and S1P receptors (S1PRs). Moreover, the mechanisms of how dysregulations of this machinery cause bone diseases, are covered.

**Expert opinion**: In the context of bone diseases, pharmacological interference with sphingolipid machinery may lead to novel directions in therapeutic strategies. Implementation of knowledge derived from *in vivo* animal models and *in vitro* studies using pharmacological agents to manipulate the S1P/S1PRs axes suggests S1PR2 and S1PR3 as potential drug targets, particularly in conjunction with technology for local drug delivery.

## 1. Introduction

**Figure 1.**
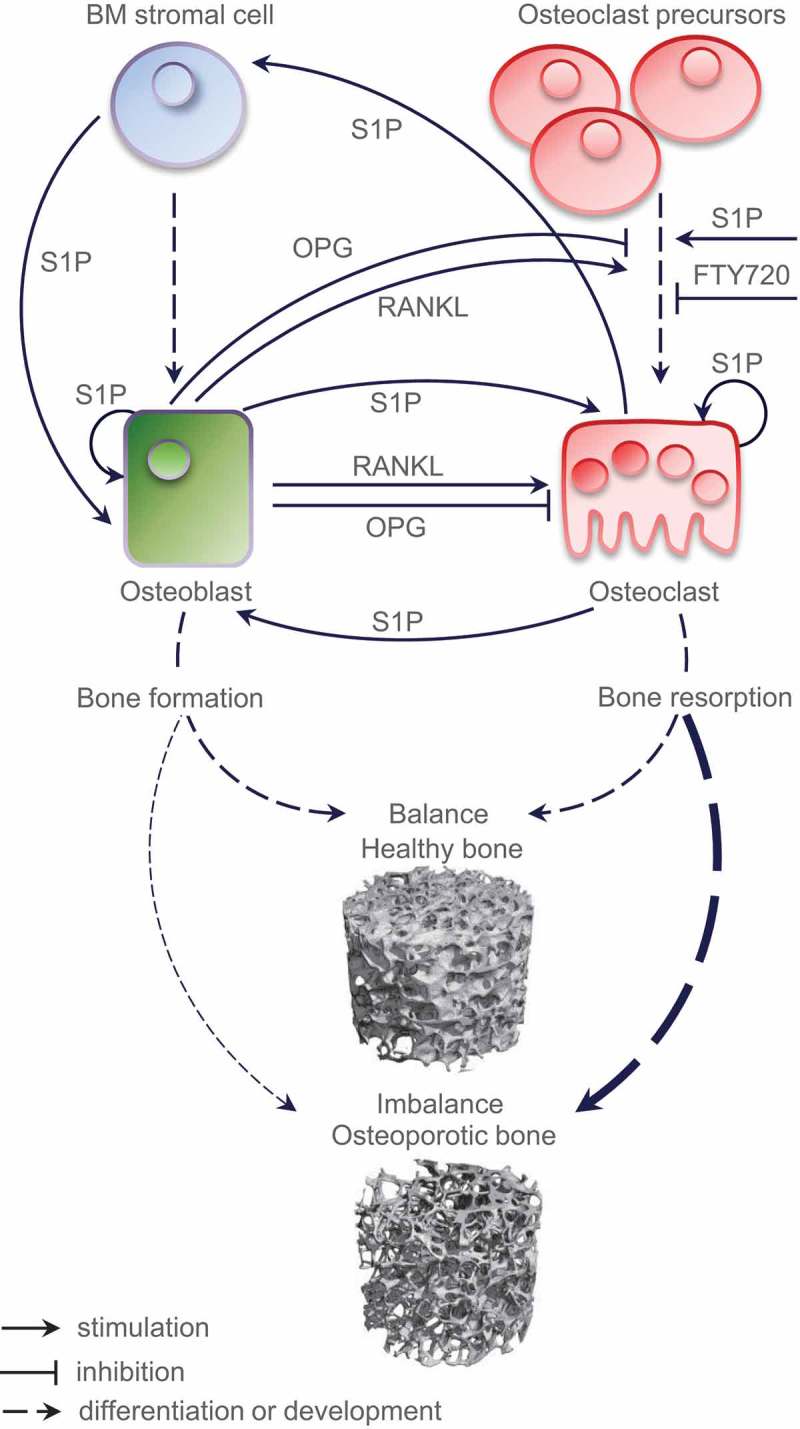
A partly hypothetical model of the role of S1P as a coupling factor in bone homeostasis. Under physiological conditions, normal bone remodeling is maintained by the balance between bone formation and bone resorption; an imbalance causes aberrant bone metabolism and leads to pathological disorders such as osteoporosis; the respective trabecular microstructures assessed by µ-computed tomography are shown. BM, bone marrow; RANKL, Receptor activator of nuclear factor-kappa B ligand; OPG, osteoprotegerin; S1P, sphingosine 1-phosphate; FTY720, the S1PRs modulator.

Cellular participants of bone homeostasis exhibit tightly regulated functional interconnections and include bone and marrow cells as well as non-osteogenic cell populations contributing through the blood supply. The major cell types regulating bone remodeling are osteocytes, bone-lining cells, osteoclast precursors (OPs), osteoclasts, osteoblasts, adipocytes, fibroblasts, megakaryocytes, and immune cell subsets including T cells and B cells [1–8]. Such cell-type heterogeneity, on one hand, ensures functional diversity and physiological reliability for bone and marrow, on the other hand, may represent a basis for anomalous signaling or dysregulation of checkpoints that affect the bone or marrow. These checkpoints could then be considered for targeting with novel therapeutic approaches. Among cytokines and chemokines as critical bioactive mediators of bone homeostasis, a central role is given to the RANK/RANKL/OPG axis [9]. Recent insights into bone biology additionally highlight the decisive and multifaceted roles of sphingolipid mediators (Figure 1).

Continuous bone remodeling ensures the quality and strength of the skeleton, preserves skeletal size and structural integrity, and secures the repair of structural microdefects. Bone remodeling occurs and takes place simultaneously and asynchronously at multiple sites known as organized bone multicellular units where clusters of bone-resorbing osteoclasts (10–20 cells) and bone-forming osteoblasts (1000–2000 cells) get instructed to act in a highly coordinated manner. Under physiological conditions, bone loss via osteoclast-mediated bone resorption is directly followed by bone replacement through osteoblast-mediated bone formation – a phenomenon known as coupling, which ensures that the bone structure is preserved [10] (Figure 1). The loss of this balance is directly linked to aberrant bone metabolism in pathological conditions such as osteoporosis, rheumatoid arthritis, periodontitis, osteolytic bone metastases, Paget’s disease of bone, and osteopetrosis [11–13]. Importantly, unbalanced bone remodeling may lead to mutually opposite aberrations, associated with either excessive loss or gain of bone mass. Osteoporosis, by way of example, is characterized by low bone mass and decreased bone strength; patients with osteoporosis are at high risk of fragility fractures of the vertebrae or hip. In contrast, bone diseases such as Paget’s disease or osteopetrosis are characterized by high bone mass, while, remarkably, bone strength is also decreased [14–17].

The physiological balance between bone resorption by osteoclasts and bone formation by osteoblasts – the key activities orchestrating continuous bone remodeling – is tightly regulated by several mechanisms. Among the critical mechanisms are (i) the programmed differentiation of osteoclasts (cells derived from hematopoietic stem cells of monocyte/macrophage lineage) and osteoblasts (cells originated from multipotent stromal stem cells), (ii) the regulated migration and trafficking of cells, including the migration of OPs between the bone marrow and blood, and the regulated migration of osteoblast precursors/osteoblasts to the resorption site, and (iii) the controlled proliferation and survival of osteoblasts, osteoclasts and their precursors.

This review discusses physiological bone remodeling in the context of emerging interrelations with the sphingolipid machinery, consisting of natural bioactive sphingolipid mediators, as well as lipid-specific G protein-coupled receptors and a set of lipid transporters. Particular emphasis is given to the cellular and molecular mechanisms linked to sphingosine 1-phosphate (S1P) as a coupling factor. Given the significant progress made in understanding sphingolipid pathobiology in several diseases, particularly in chronic inflammation, immune disorders, and cancer, sphingolipid-related checkpoints emerge as attractive targets. Compelling yet puzzling evidence from certain areas of bone research also proposes those critical checkpoints to be considered for therapeutic intervention(s). Considerable work, however, remains to be done in understanding the multifaceted roles of the complex sphingolipid machinery in the pathobiology of bone disorders.

## 2. The biology of S1P

Among the plethora of sphingolipid metabolites, S1P has emerged as a potent mediator with diverse effects on multiple biological processes including proliferation and survival, cytoskeletal organization and migration, adherence and tight junction assembly, and morphogenesis. S1P is a constituent of serum and plasma (in high nanomolar to low micromolar concentrations), where it is bound to carrier proteins, such as serum albumin, high-density lipoproteins, and oxidized low-density lipoproteins [18,19]. Intriguingly, S1P can be found in normal tissues as well at low concentrations. This natural lipid gradient belongs to the strongest chemoattractant system for certain cell types; the S1P-attributed trafficking of hematopoietic/immune cells *in vivo* is the best-studied example. The S1P gradient is also utilized by bone cells as a migratory signal and will be discussed later. A peculiarity of S1P as a signaling molecule lies in its dual mechanism of action, since it may act as a second messenger within cells and, when secreted from cells, as an extracellular mediator acting in an autocrine and/or paracrine manner via binding to five specific G-protein-coupled receptors (S1PR1-5, formerly EDG1, 3, 5, 6, and 8) with affinities within low nM ranges. The first S1PR (previously EDG1, endothelial differentiation G-protein-coupled receptor 1) was identified in 1998 and described as an early gene from endothelial cells; the whole S1PR family has been best characterized in the vascular and the immune system [20–22]. The current body of evidence suggests that there is no cell type which does not express S1P receptors in any repertoire, which in turn indicates that all cells are responsive to S1P in some manner. Cells participating in bone homeostasis are also S1P sensitive; the progress made in understanding the role of S1P/S1PRs axes in bone turnover will be discussed in the following chapters. It should be noted that the complexity of S1P-mediated biological effects is not restricted to the expression pattern of its specific receptors and should be considered in a broader context with S1P-producing and S1P-degrading cellular enzymes and their varying cell type-specific regulations.

**Figure 2.**
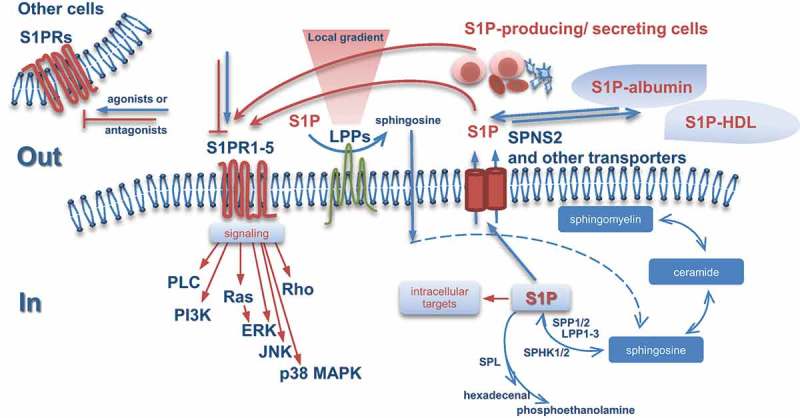
Interconnected processes of (i) S1P synthesis and degradation within the sphingomyelin/salvage pathway, (ii) S1P export and (iii) signaling through binding to five specific G-protein-coupled receptors, S1PR1-5. S1P can function as an autocrine, intracrine, paracrine, or endocrine bioactive mediator. Both osteoblasts and osteoclasts are among the cells which are able to produce, secrete, and respond to S1P. S1P, sphingosine 1-phosphate; S1PR, sphingosine 1-phosphate receptor; SPHK, sphingosine kinase; SPP, sphingosine-1-phosphate phosphatase; LPP, lipid phosphate phosphatase; SPL,sphingosine-1-phosphate lyase; SPNS2, spinster homolog 2; HDL, high-density lipoprotein; PLC, phospholipase C; PI3K, phosphoinositide 3-kinase; Ras, rat sarcoma; Rho, ras homolog; ERK,extracellular-signal regulated kinase; JNK, c-Jun N-terminal kinase; MAPK, mitogen-activated protein kinase.

While synthesis of S1P is catalyzed by two sphingosine kinases (SPHK1 and SPHK2) [23], its degradation is controlled by two different classes of enzymes – via irreversible cleavage by S1P lyase (SPL) [24,25] and via dephosphorylation by the S1P-specific phosphohydrolase family members, SPP1 and SPP2 [26–28] (Figure 2). Additionally, a more general degradation pathway through dephosphorylation via members of the broad specificity lipid phosphohydrolase family (LPPs) also exists; consistent with the plasma membrane localization and the proposed structure of the active site, LPPs are believed to function predominantly as ‘ecto-phosphohydrolases’ converting/deactivating extracellular S1P [29,30]. The major physiological roles of LPP family members are not fully defined; there might be non-exclusive mechanisms for (i) the clearance of extracellular S1P (as well as other phosphorylated forms of lipids such as lysophosphatidic acid, LPA, and ceramide 1-phosphate, C1P) leading to changes of the local lipid gradient close to membrane receptors and thereby regulating signaling events and/or (ii) the subsequent intracellular accumulation of dephosphorylated lipid mediators, including sphingosine (as well as monoacylglycerol and ceramide) which can modulate other intracellular signaling routes and/or be converted back to S1P. Undoubtedly, the repertoire of S1P-driven biological outcomes will have cell type-specific characteristics based on (i) the cell type-specific expression pattern of S1P receptors, (ii) their differential coupling to heterotrimeric G-proteins, which can activate multiple signaling cascades, (iii) the cell type-specific expression signature of S1P-producing versus S1P-degrading enzymes, and (iv) the cell type-specific expression pattern of currently known S1P transporters ensuring the S1P transport to the extracellular environment. Another important aspect affecting local S1P concentrations is the local availability of S1P-producing/secreting cells. Production of S1P is well documented for activated platelets, erythrocytes, and other non-hematopoietic cells such as vascular and lymphatic endothelial cells; synthesis and secretion has been reported for mast cells, neutrophils, and epithelial cells of different origin [19,31,32]. This suggests that the microenviroment within a particular tissue or tissue compartment will impact or even define the S1P levels and the spectrum of S1P effects. Regarding bone and marrow, it is important to consider that erythrocytes, as one of the main cellular factories producing S1P, are generated in bone marrow and may play a very special, yet unknown role in the S1P/S1PR-attributed mechanisms underlying bone homeostasis. Furthermore, during the early phase response [33], bone injury is accompanied by a local platelet activation and platelet-mediated secretion of a platelet-derived growth factor (PDGF) and likely S1P. Osteoclasts and osteoblastic cells also secrete substantial amounts of S1P within the bone microenvironment [34,35].

The biological outcomes attributed to modulated S1P levels can be summarized as follows: (i) increased intracellular S1P shifts the cellular status to a pro-survival/anti-apoptotic phenotype; (ii) decreased S1P leads to reduced proliferation, that in combination with other mediators, might reinforce cell differentiation processes; (iii) aberrant regulation of any enzyme producing or utilizing ceramide may modify the S1P/ceramide cellular rheostat and thereby affect survival/apoptosis and/or trigger perturbations in membrane lipid composition affecting lipid rafts and signaling; (iv) the local S1P gradient between marrow tissue and blood and/or the gradient created by a cell as a result of local S1P degradation via enhanced action of membranous S1P phosphatases acts as a crucial dominant factor underlying the directed cell migration as described for other cell populations and other tissues [36,37]; (v) our recent work interconnects S1P as well as other participants of the cellular sphingolipid machinery with the pro-survival, pro-migratory properties of cells undergoing the pathological epithelial to mesenchymal transition program and thus points to their contribution to more general mechanisms underlying cell plasticity and cell fate decisions [38].

## 3. FTY720 as a sphingolipid mimetic

Strong evidence indicates that sphingolipid-related targets as well as sphingolipid analogs may have a great potential for treatment of various diseases. One example is FTY720 [2-amino-2-[2-(4-octylphenyl)ethyl]propane-1,3-diol hydrochloride] (also named fingolimod, trade name Gilenya™, Novartis) that was approved by the FDA in 2010 for the treatment of relapsing forms of multiple sclerosis [39]. FTY720 is a mimetic of natural sphingosine and therefore can be recognized by part of the cellular sphingolipid enzymatic machinery [40,41]. Upon *in vitro* and *in vivo* phosphorylation by SPHK type 2, the resultant phosphorylated form, FTY720-P, acts as a mimetic of S1P and specifically binds to four out of five S1PRs with one exception being S1PR2 [18,42]. The drug is a unique immunomodulator; the mechanism of action has been predominantly attributed to sequestration of circulating lymphocytes in primary and secondary lymphoid organs/tissues. Uniquely, FTY720-P has a dual agonistic/antagonistic mode of action; although FTY720-P acts as an agonist at S1PR1/3/4/5, in the longer term its effects are inhibitory on S1PR function as best documented for S1PR1 in lymphocytes. The mechanism of functional antagonism was suggested to be linked to receptor internalization and in part is based on the ability of FTY720-P to target the S1PR1 receptor to the proteasomal degradation pathway through poly-ubiquitination [43]. Additionally, in contrast to S1P, which can be irreversibly cleaved by SPL, FTY720-P is resistant to SPL action. This aberrant receptor internalization may render lymphocytes unresponsive to natural S1P, representing an obligatory signal for lymphocyte recirculation between lymphoid organs and blood [44]. Supporting S1PR1 desensitization due to the internalization process, administration of a specific S1PR1 antagonist (SEW2971) in an animal model resulted in decreased S1P-mediated cell migration [45]; this is furthermore strongly supported by the experimental outcome in knock-in mice with a mutated C-terminal, serine-rich S1PR1 motif, which plays an important role in internalization of the S1PR1 receptor [46].

There are, however, other properties of FTY720 which are independent of its phosphorylation status and binding to S1PRs and are often neglected during data analyses. Yet, this is an important consideration, since upon FTY720 administration *in vivo* both the parental FTY720 and FTY720-P can be detected and the steady state between non-phosphorylated and phosphorylated drugs in the blood/serum is reached within 1 h [47]. The non-phosphorylated form of FTY720 is taken up by a cell similarly to natural sphingosine [41] and thus may affect multiple pathways by targeting intracellular molecules (reviewed in [48]); interestingly, FTY720 modulates the activity and expression of SPHK1 [49]. Furthermore, LPP3 functions as an *ecto*-phosphatase for FTY720-P thereby regulating levels of FTY720-P and FTY720 in close proximity to the plasma membrane as well as the follow-up uptake of non-phosphorylated FTY720 by a cell. Thus, together with SPHK2, LPP3 determines the extracellular/intracellular ratio between FTY720-P and FTY720 [41]. An interesting finding is that the presence of even few LPP3^high^-expressing cells within a mixed cell population is sufficient to drive local FTY720-P conversion to non-phosphorylated FTY720 enabling FTY720 uptake by various cells in the vicinity, even in those with low intrinsic LPP3 levels/activity.

The complexity and the peculiar properties of FTY720/FTY720-P as the biological system should be taken into account upon *in vitro* and *in vivo* applications of either form of the drug and subsequently, for interpretation of the output data in the context of the cell-type specificity.

Overall, the success of this drug is considered as a ‘proof of concept’ for the potential exploration of other sphingolipid-related targets for a wide spectrum of diseases. Studies involving FTY720 have considerable importance for understanding the regulation of cell motility as well as the transition from proliferation to differentiation, which contribute to immunity and beyond, including physiological and pathological bone remodeling.

## 4. S1P and osteoclastogenesis

According to the current state of knowledge, the SPHK1/S1P axis in osteoclastogenesis (mouse studies) has a double-edged role; the microenvironment seems to play a decisive role. On one hand, SPHK1 negatively affects differentiation when osteoclasts are generated from bone marrow-derived macrophages (BMMs) in a single cell culture in the presence of RANKL and M-CSF. On the other hand, SPHK1 and the SPHK1-produced S1P potentiate osteoclastogenesis under conditions when BMMs are cocultured with osteoblasts [34]. In the first case, osteoclast differentiation was accompanied by an upregulation of SPHK1/2 on both mRNA and protein levels and associated with increased intracellular levels of S1P in differentiated osteoclasts. Although both SPHKs were upregulated, based on gain-/loss-of-function experiments, involvement of only SPHK1 in RANKL-driven osteoclast formation has been proposed. Furthermore, in contrast to the TNFalpha-mediated SPHK1 activation via TRAF2 [50,51], the RANKL-driven triggering of SPHK1 activity seems to act through the formation of a SPHK1/TRAF6 complex [34]. Contrary to expectations, an inverse correlation between SPHK1 activity and the process of osteoclastogenesis was shown. Thus, silencing of SPHK1 resulted in enhanced osteoclastogenesis via RANKL-mediated augmentation of p38 MAPK activity paralleled by reduction in ERK1/2 MAPK activity; the signaling process was further accompanied by upregulation of c-Fos and NFATc1 levels in a p38-dependent manner. Notably, exogenously added S1P did not have a negative effect on osteoclast differentiation, indicating that intracellular rather than extracellular S1P might be responsible for the observed effects [34]. In the second case, osteoclast generation was potentiated by exogenous S1P when added to cocultures of BMMs with osteoblasts in the presence of 1α,25-dihydroxyvitamin D_3_ (VitD_3_) and/or low concentration of prostaglandin E_2_ (PGE_2_). Osteoblasts treated with S1P showed an increase in RANKL mRNA expression levels accompanied by a decrease in OPG mRNA levels, resulting in further increase of the RANKL/OPG ratio. The S1P-dependent RANKL upregulation was mediated via the p38 ERK and, to a lesser extent, via JNK MAPKs, which in sum trigger PGE_2_ through COX2 activation. Intriguingly, an addition of FTY720, the parental non-phosphorylated drug, to the cocultures of BMMs and osteoblasts showed strong inhibitory effects on osteoclastogenesis [34]. The authors interpret this finding as additional supporting evidence for the role of S1P/RANKL axis in osteoclast – osteoblast coupling. Nonexclusively, the data might additionally point out the contributing, as yet uninvestigated role of SPHK type 2, being responsible for intracellular FTY720 phosphorylation, in the process of osteoclast differentiation. There, might also be direct intracellular target(s) of FTY720 involved.

Taken together, this reciprocal interconnection between SPHK1/S1P and RANKL including the S1P-COX2-PGE2-RANKL pathway might represent a novel critical axis in normal bone remodeling. Under pathophysiological circumstances, the SPHK1/S1P-attributed mechanisms might be considered as novel checkpoints contributing to bone degradation.

## 5. S1P-dependent mobilization of OPs on the bone surface

### 5.1. S1P/S1PR1 axis

**Figure 3.**
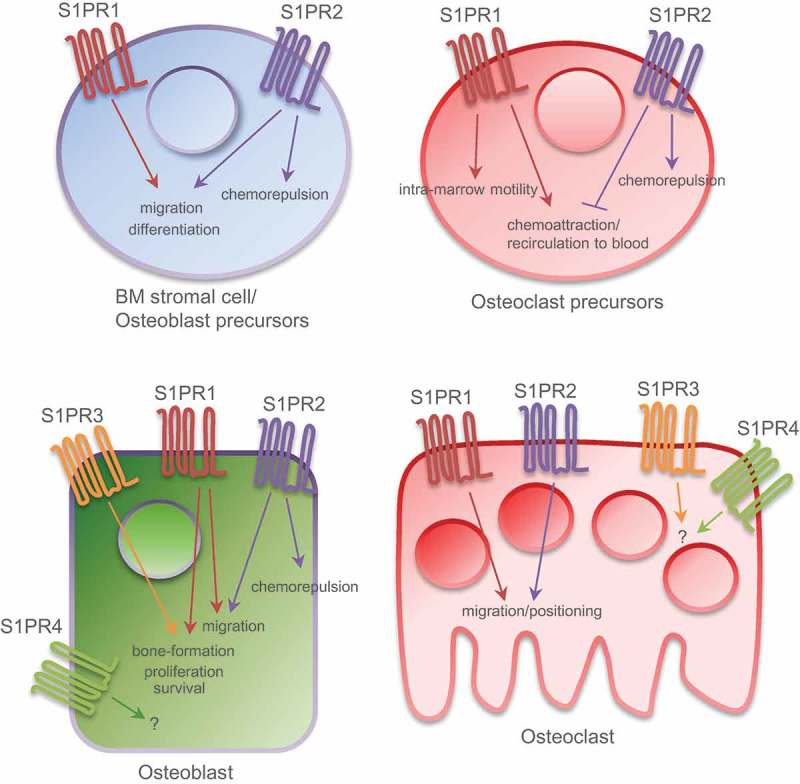
Expression pattern of S1PRs on bone cells and their downstream cellular responses. This illustration summarizes the current knowledge derived from studies/cells of mouse, rat and human origin. Osteoblast precursors and osteoclast precursors express S1PR1 and S1PR2; osteoblasts and osteoclasts express S1PR1-4. Which S1PRs underlie the pro-survival and proliferative effects, however, was not yet evaluated. Implementation of knowledge allows to consider S1PR2 and S1PR3 as potential drug targets in bone pathobiology.

Although the picture is by far not complete, several cytokines including the prominent CXCL12 (SDF-1) have been proposed to regulate osteoclast migration [52–54]; yet little is known about the exact mechanisms that fine-tune the residence stability of OPs on the bone surface where they acquire the fully differentiated cell state and perform their bone resorbing function. It is now clearly established that the S1P gradient and the S1P/S1PRs axes play a crucial role in the migration of various subsets of immune cells under homeostatic and disease conditions [18,55,56]. This argues strongly in favor of a similar impact of the sphingolipid machinery in controlling cell migration in the course of bone remodeling. Indeed, mouse OPs express two out of the five known cell surface receptors that recognize S1P, namely S1PR1 and S1PR2 [57,58] (Figure 3). Accordingly, OPs exhibit a positive chemotactic response to S1P when an S1P gradient was established *in vitro* in a cell-based model. Importantly, the S1P-driven effect was also confirmed *in vivo* in mouse models using two-photon imaging of calvaria bone tissue. Intravenous application of a selective agonist of S1PR1, SEW2871 [59], to two strains of mice where subsets of myeloid cells were expressing EGFP [60,61], stimulated the motility of BM-resident OP-containing monocytoid populations with some mobilized cells entering the blood circulation [58]. Interestingly, the S1P/S1PR1-driven intra-marrow motility of OPs and their RANKL-driven differentiation seem to represent two temporally mutually exclusive processes as the exposure of OPs to RANKL causes the NFκB-dependent downregulation of S1PR1 on the cell surface thus preventing the S1PR1-dependent response to S1P [58].

To analyze S1P effects in bone homeostasis in more detail, Masaru Ishii et al. [58] generated mice deficient in S1PR1 specifically in osteoclasts/monocytes (cS1PR_1_^−^^/^^−^). In those animals, the overall bone tissue density was significantly impaired and was accompanied by a decrease in both trabecular thickness and trabecular density, demonstrating an osteoporotic state. The authors furthermore observed an increase in the osteoclast attachment ratio in cS1PR_1_^−^^/^^−^ mice, calculated as bone surface occupied by osteoclasts normalized to total bone surface. Since a direct effect of S1P on RANKL-driven osteoclast differentiation in the *in vitro* system was not observed, this suggested that the observed phenomena are due to the loss of S1P-mediated chemotactic behavior of OPs. Given that the S1P concentration is known to be lower in tissue that in blood [62] thus creating an S1P gradient, the data further suggested the decreased recirculation of OPs from bone tissues to blood in cS1PR_1_^−^^/^^−^ mice, leading to their accumulation at the bone surface followed by formation of proresorptive mature osteoclasts. These conclusions, however, do not take into account the spatial-temporal interconnection with bone-forming osteoblasts.

Generally, under physiological conditions, S1P-mediated chemoattraction has to be kept under strict control to ensure stable localization and maturation of OPs on the bone surface. To prevent S1P-driven recirculation of premature osteoclasts to the blood, the cellular machinery represses S1PR1 expression in response to RANKL [58]. The obtained findings highlighting the impact of the S1P/S1PR axis are of particular interest for drug-targeting strategies, since the signaling and downstream pathways can be interfered with by multiple sphingolipid-related routes, including FTY720 treatment (reviewed in [63]). Indeed, FTY720 administration prevented bone loss after ovariectomy in mice [58]; ovariectomy-induced osteoclast deposition on the bone surface was in part restored to the normal physiological level due to enhanced migration of OPs back to circulation. Furthermore, the short-term mobility change was even more pronounced upon treatment with SEW2871 [59], the S1PR1 agonist indicating that, in this context, FTY720 likely acts as a functional agonist. The authors however could not exclude the contribution of other S1P-responsive cell types, in particular immune cells. Taken together, the findings nominate the S1P/S1PR axis as a critical checkpoint in osteoclastogenesis, and propose for consideration novel therapeutic options to interfere with pathological bone remodeling.

### 5.2. S1P/S1PR2 axis

What is known about the functional role of the S1PR2, expressed on OPs? The conclusion that S1PR2 affects OPs mobilization was originally based on two observations from *in vitro* cell-based chemotactic models (mouse cells, RAW264.7 and BM-derived M-CSF-dependent monocytes). The first finding demonstrated that the chemoattractant property of S1P is concentration dependent and shows bell-shaped characteristics: strong S1P-mediated chemoattraction occurs at S1P concentrations <10^–7^ M and is less pronounced at higher S1P concentrations [57]. The second finding described how the silencing of S1PR2 via siRNAs triggers enhanced cell migration at high S1P concentrations, suggesting that S1PR2 deficiency increases S1P/S1PR1-driven chemotaxis [57]. *In vivo*, two-photon imaging using mouse models with EGFP-labeled OPs confirmed this conclusion, as application of the S1PR2 antagonist JTE013 [64] resulted in increased motility of CX_3_CR1-EGFP-positive cells in BM space [57,65]. In summary, the data suggested that the fine balance between S1PR1 and S1PR2 expression and activity impacts the migratory behavior of OPs.

Novel evidence was obtained using S1PR2-deficient mice [66]. S1PR2^−^^/^^−^ mice showed higher bone density accompanied by decrease in osteoclastic bone resorption thus showing overall moderate osteopetrosis as compared to control littermates [57]. The next important question is whether interfering with S1PR2 function using specific receptor antagonist(s) will demonstrate a therapeutic potential in the course of osteoporosis. *In vivo* administration of the S1PR2 antagonist JTE013 [64] limited osteoclastic bone resorption and reversed bone density loss in the RANKL-induced osteoporosis mouse model [67], and improved bone parameters such as bone matrix density, trabecular thickness, and trabecular density in the conventional model for postmenopausal osteoporosis in ovariectomized mice [57]. Taken together, according to a current model, the trafficking and mobilization of OPs in bone tissue is controlled by the coordinated interplay between the reciprocally acting S1P/S1PR1 and S1P/S1PR2 axes and is largely based on the S1P gradient established between blood (high S1P) and bone marrow tissue (low S1P) [68]. According to this model, S1PR2 requires higher exogenous S1P concentration for activation and thereby OPs are attracted to bone by the action of the S1P-driven chemorepulsion/negative chemotactic response via S1PR2; conversely, OPs could re-enter blood circulation following S1PR1-mediated chemoattraction [57].

There are, however, a few limitations of the proposed model, which should be mentioned. (I) The impact of other chemoattractants and adhesion molecules regulating both the entry and exit routes of OPs have not been evaluated in the context of the modulation of S1P/S1PRs axes. (II) Bone is a complex multicellular organ, where the tight functional interconnections between distinct cell populations take place in both a temporal and a spatial manner; the modulation of S1P receptors using specific antagonists will affect the behavior of all S1PR1- or S1PR2-expressing cells, including various immune cell subsets (relevant for both receptors) and endothelial cells (predominantly S1PR1). The impact of either cell type to the orchestration of OPs cannot be excluded. (III) The tight balance between chemoattraction and chemorepulsion of OPs might be further impacted by the local spatial/temporal differences in S1P levels created within the marrow by S1P-secreting cells, particularly considering erythrocytes (one of the main known S1P factories), which are produced in the BM [69]. Furthermore, the local S1P gradient can be created by various cell types based on the action of lipid *ecto*-phosphatases [30], though information on the cell type-specific expression patterns and regulation of activities within marrow is very limited. (IV) We also have limited knowledge regarding the S1PR repertoire at various stages of octeoclasto/osteoblastogenesis; consequently, other S1P receptors might come into play and be considered as additional drug targets for pathological bone remodeling.

Generally, despite the gaps in our understanding, the regulation of OPs trafficking/recruitment via modulation of S1P-mediated events might be considered as a future therapeutic strategy in bone disorders such as osteoporosis. This would be principally different to the mode of action of the current standard regimen of blocking the mature osteoclasts, as with bisphosphonates.

### 5.3. S1P/S1PR2 axis and vitamin D

Vitamin D is a well-known critical factor regulating calcium homeostasis and bone resorption [70], yet, the underlying mechanisms are not fully understood. A recent study by Kikuta et al. [71] identifies the S1P/S1PR2 axis as an important factor linking the anti-resorptive action of active vitamin D and the migratory behavior of circulating OPs. More precisely, calcitriol [1α,25(OH)_2_D_3_] and its therapeutic analog eldecalcitol were shown to moderately downregulate S1PR2 expression in RAW264.7 cells, a mouse macrophage/monocyte lineage cell line, whereas the expression of S1PR1 and the chemokine receptors CXCR4 and CX_3_CR_1_ were not affected. In line with the results obtained from the study using S1PR2-knockdown RAW264.7 cells [57], the enhanced migration of cells toward higher S1P concentrations was observed *in vitro* in response to preincubation with calcitriol or eldecalcitol. *In vivo*, oral administration of calcitriol or eldecalcitol, known to diminish bone loss in ovariectomized mice [72,73], resulted in moderate downregulation of S1PR2 on CD11b^+^ OPs [71]. Furthermore, using the CX_3_CR1-EGFP knock-in mouse model described above and applying the multiphoton bone microscopy technique [57,58], migration of CX_3_CR1-EGFP^+^ OPs was monitored and found to be increased upon treatment with calcitriol or eldecalcitol [71], in line with the results obtained for the S1PR2 antagonist [57].

Besides describing novel functions of vitamin D in bone remodeling, this study provides additional evidence supporting the idea that downregulation of S1PR2 expression is associated with reduced osteoclastic bone destruction. There are, however, two critical points to consider: (i) whether moderate downregulation of S1PR2 mRNA levels (about 30%) alone is sufficient to explain the observed biological effects, and (ii) whether the heterogeneity of CD11b^+^ cells should be considered for data interpretation, as they are isolated from the spleen and bone marrow and used under the assumption that they contain a high number of circulating monocytoid cells.

## 6. S1P/S1PR1 and S1P/S1PR2 axes in the regulation of migration of osteoblast precursors

Apparently, the S1P-attributed effects on bone homeostasis are not limited to osteoclasts. In normal bone turnover, osteoclast-mediated resorption is tightly coupled to osteoblast-mediated bone formation, and multiple data clearly demonstrate that osteoblasts direct osteoclast differentiation [74]. Within the feedback loop mechanisms, are there novel osteoclast-derived factors promoting either the directed migration of osteoblast precursors/osteoblasts to the resorption site and/or their developmental program? Given the fact that conditioned media from osteoclasts is known to direct osteoblast recruitment and maturation, an attempt was done by Pederson et al. [75] to identify the spectrum of novel powerful coupling factors with a focus given to secreted mediators. Upregulated SPHK1 was among the modulated genes in mature osteoclasts compared with precursors, suggesting that SPHK1-produced S1P would be also elevated and biologically active. Indeed, the addition of S1PR1 antagonist VPC 23019 together with the osteoclast-produced conditioned media to human mesenchymal stem cells attenuated both the random movement/chemokinesis and mineralization of the mesenchymal stem cells, thus nominating the lipid mediator S1P as novel coupling factor [75]. A more recent study [76] also documented the S1P chemoattractive response in mesenchymal cells and further showed that pharmacological manipulation of S1P/S1PR axes with agonists or antagonists leads to increased or reduced migratory capacity, respectively. In osteoblast precursors, S1P-mediated migration is promoted by both receptors via JAK1/STAT3 for S1PR1 and FAK/PI3K/AKT for S1PR2. Intriguingly, besides its contributing role in cell migration, S1PR1 expression was found to be upregulated during the bone morphogenetic protein 2 (BMP2)-driven transition from mesenchymal progenitors to mature osteoblasts and could thereby be nominated as a novel marker of osteoblastogenesis [77]. These findings additionally suggest a stage-characteristic response to S1P.

Furthermore, with respect to chemotactic activity, there is cross talk between S1P and PDGF signaling [78]. The directional chemotaxis of preosteoblasts (murine MC3T3-E1 cells) toward PDGF was inhibited in the presence of S1P, while the mature osteoblasts were insensitive to S1P. At the same time, the random movement, known as chemokinesis, of neither preosteoblasts nor osteoblasts was affected by S1P. Using elegant *in vitro* experimental settings, the authors additionally conclude that, under certain circumstances, S1P may act as a chemorepellent driving cell migration against its own gradient. Of note, pre-osteoblasts were found to express two out of five S1PR types (S1PR1 and S1PR2; Figure 3); upon BMP2-driven maturation, the S1PR2 expression is downregulated [78]. This suggests an involvement of S1PR2 to the stage-dependent cellular response to S1P. Interference with S1PR2 signaling/activity by both the selective antagonist JTE-013 [79] and the RNA interference strategy confirmed the role of S1PR2 in S1P-driven chemorepulsion. Furthermore, the constitutive S1PR2 expression preserves sensitivity to S1P both at the preosteoblast and the osteoblast stage [78]. Overall, the data suggest that the expression and/or activity of S1PR2 is a critical decisive factor(s) for the response of osteoblasts to S1P ensuring the scenario under which the osteoblast precursors are preserved in the marrow during osteoblastogenesis. Interestingly enough, as we discussed above, the same S1P/S1PR2 axis impacts the ability of OPs to migrate against an S1P gradient established between the bloodstream and the bone marrow.

## 7. S1P in proliferation and survival of osteoblasts

An additional effort was made in order to determine the role of S1P on proliferation and survival of osteoblasts; moreover, in some studies the outcome effects were compared for S1P and the related lysophospholipid LPA. In primary rat osteoblasts, S1P was found to exert mitogenic effects predominately via functional G*i* proteins and p42/44 MAP kinases [80]. An earlier study by the same authors demonstrated an increased osteoblast proliferation in response to LPA [81]. Targeting PKC isoforms with specific antisense oligodeoxynucleotides in primary human osteoblastic cells *in vitro* demonstrated that S1P-driven proliferation is predominantly linked to the activity of PKCalpha [82,83]. Given that bone remodeling is known to be associated with substantial loss of osteoblasts by apoptosis (up to 65%) [84], attention was paid to the potential impact of S1P (and LPA) on osteoblast survival [85]. Both bioactive lysophospholipids were found to decrease apoptosis in serum-deprived conditions in primary rat osteoblasts as well as in human osteoblastic cell line SaOS-2. The S1P- and LPA- triggered pro-survival effects were found to act via G*i* proteins and downstream signaling by Pi-3 kinases. In contrast to the mitogenic effects, which were found to be p42/44 MAP kinase dependent [80], the anti-apoptotic mechanisms did not require p42/44 MAP kinase activation [85]. Which S1PRs underlie the observed mitogenic and anti-apoptotic/pro-survival effects, however, has not yet been evaluated. Overall, the data further support the multifaceted role of S1P in the regulation of complex, multilayer mechanisms of bone metabolism, and strongly emphasize the necessity to consider the interconnection between the sphingolipid turnover and signaling and LPA signaling, and to expand our current understanding of the LPA-associated pathways during bone homeostasis (comprehensive overview of LPA and bone homeostasis is summarized in [86,87]).

## 8. Additional S1P-attributed mechanisms regulating the bone resorption – bone formation rheostat

Several recent studies have emphasized the potential of S1P as a critical osteoanabolic factor [88,89]: S1P was identified as a key osteoclast-derived coupling messenger that locally promotes osteoblasts’ bone-forming capacity. Novel insights into coupling mechanisms were highlighted in studies addressing the effect of Cathepsin K on bone formation. Cathepsin K is a protease released by osteoclasts and essential for degradation of matrix proteins including collagen [90]. Targeted deletion of *Ctsk*, the gene encoding this cysteine protease, in murine hematopoietic cells – more specifically, in osteoclasts – resulted in impairment of bone resorption and an increase in bone formation, providing additional experimental evidence for the existence of a coupling mechanism *in vivo*. Intriguingly, SPHK1 mRNA and protein levels were increased in osteoclasts generated from *Mx1;Ctsk^fl/fl^* and *CD11b;Ctsk^fl/fl^* mice. A significant increase of secreted S1P was consequently observed in osteoclast-conditioned medium, and this medium in turn stimulated alkaline phosphatase activity and mineralization in CD1 calvarial osteoblasts in a S1PR1/3-dependent manner, as determined by applying VPC23019, an S1PR1/3 antagonist [88].

A different aspect of S1P – acting as a clastokine and coupling factor – was highlighted by Keller et al. in a study addressing the impact of calcitonin on bone remodeling [89]. The hormone calcitonin is a known calcium-lowering factor, exerting its effect via the calcitonin receptor, and acting as an inhibitor of bone resorption [91]. However, in contradiction with the clearly defined pharmacologic mode of action, in patients with long-term excess of calcitonin due to medullary thyroid cancer bone mineral density is normal [92]. In line with physiological observations, the cell type-specific deletion of calcitonin receptor in mouse osteoclasts resulted in an increase in trabecular bone volume; furthermore, S1P release by osteoclasts was enhanced [89]. On the basis of genome-wide expression analysis, a linkage between calcitonin and *Spns2*, the gene encoding the S1P- specific transporter, was discovered. As discussed above, SPNS2 expression is among the critical factors determining the extracellular levels and thus the biological activity of S1P molecule acting in an autocrine and/or paracrine mode. Interestingly, the skeletal phenotype of calcitonin receptor-deficient mice having increased bone mass was normalized by deletion of *S1pr3* emphasizing the biological relevance of the S1P/S1PR3 axis in the osteoanabolic activity of S1P. In support, administration of FTY720 resulted in increased bone formation as measured by increased trabecular bone volume in wild-type mice, but not in *S1pr3*-deficient mice [89]. Although the contributing role of other S1P receptors cannot be completely excluded, these data demonstrate that S1PR3 is necessary to promote bone formation in response to S1P. Hence, the authors discuss the possibility of using selective S1PR3 agonists as a novel promising therapeutic approach for osteoporosis. The findings of this study once again emphasize the key role of S1P in bone remodeling and expand our knowledge of sphingolipid-related druggable checkpoints controlling the cross talk between osteoclasts and osteoblasts.

## 9. Clinical studies linking bone status and S1P levels

As discussed above, the collective findings of the investigations performed in cell-based systems and in mouse models highlight the link between S1P and the multifaceted process of bone remodeling, and are instructive for clinical studies in patients aiming to assess the association between blood S1P levels and bone status. Initial findings by Lee et al. [93] demonstrated that postmenopausal women showed higher S1P plasma levels in comparison to premenopausal women and men. Furthermore, S1P levels correlated positively with bone resorption markers and showed a negative correlation with bone mineral density values [93], strongly supporting the hypothesis that circulating S1P may impact bone homeostasis. The parallel study by Kim et al. [94], investigating the associations between plasma S1P levels and the risk of vertebral fracture, demonstrated that S1P levels were significantly lower in the group of postmenopausal women without vertebral fractures in comparison to the group with vertebral fractures; in the latter group the plasma S1P levels showed a positive correlation with the number of vertebral fractures. Notably, the statistical significance persisted when adjusted for BMD – the parameter used thus far for vertebral fracture risk prediction, however, with only limited sensitivity [95]. Given that, the authors suggested blood S1P as a novel biomarker for risk assessment of osteoporotic vertebral fracture.

## 10. Expert opinion

We have made an effort to summarize, analyze, and review the progress made in bone research evaluating the current knowledge and our current understanding of the sphingolipid-related mechanisms regulating bone remodeling (Figures 1 and 3). There are strong lines of evidence suggesting a fundamental and multistep involvement of bioactive sphingolipids in a variety of aspects of bone metabolism. One should consider that the sphingolipid system is highly complex, and that the individual players of the sphingomyelin pathway, including S1P, are strongly interconnected into a dynamic network. Under physiological conditions, the proper function of diverse sphingolipid molecules is guaranteed by tight coordination of their synthesizing, degrading, and modifying enzymes, as well as of their specific cell surface receptors and transporters – altogether building up the entire sphingolipid machinery [38,96]. Thus far, bone biology research groups have focused particularly on prominent players such as SPHKs, bioactive S1P, and the S1P receptors. More specifically, S1P can be regarded as a coupling factor responsible for osteoclast – osteoblast cross talk and as a chemotactic factor that regulates the migration of bone cells. In light of this knowledge, we propose the relevant checkpoints of the sphingolipid machinery as promising therapeutic targets for bone diseases.

Many bone diseases result from disturbances of bone remodeling whereby bone resorption exceeds bone formation. Thus, osteoporosis, rheumatoid arthritis, and osteolytic bone metastases are promising indications for sphingolipid-based therapies. Among these, osteoporosis appears to be a particularly interesting indication. Osteoporosis is by far the most frequent metabolic bone disease; it has been estimated that in the European Union 22 million women and 5.5 million men have osteoporosis [97].

As proposed by Riggs and Parfitt [98], two classes of drugs for the treatment of osteoporosis can be distinguished: anti-catabolic (decreasing bone remodeling and preserving bone microarchitecture; examples are bisphosphonates and denosumab) and anabolic (increasing bone remodeling, where bone formation exceeds bone resorption; an example is teriparatide). Regarding sphingolipid-related mechanisms in bone turnover, in our opinion two drug targets appear particularly attractive: S1PR2 and S1PR3; however, thereby opposing targeting strategies should be considered with receptor antagonist(s) for S1PR2 and receptor agonist(s) for S1PR3. A rationale for S1PR2 as a therapeutic target can be derived from the observation that S1PR2 knockout mice develop osteopetrosis. Since, as described above, S1PR2 in OPs mediates S1P-driven chemorepulsion from blood to bone, we hypothesize that pharmacologic inhibition of S1PR2 will decrease bone resorption and, act as an anti-catabolic drug, consequently increasing bone mineral density. Nevertheless, the utility of S1PR2 inhibition for the treatment of osteoporosis will depend not only on its effect on osteoclasts but also on that of osteoblasts. Having this in mind, we should like to emphasize that – in contrast to OPs (in which S1PR1 and S1PR2 have opposing effects on migration) – activation of both S1PR1 and S1PR2 may stimulate the migration of osteoblast precursors [76]. This could imply that anti-catabolic effects of S1PR2 inhibition are more pronounced in osteoclasts than in osteoblasts, a notion that is supported by the observation of a high-bone mass phenotype in S1pr2-deficient mice. A possible limitation of this approach to develop novel anti-catabolic drugs by targeting the S1P/S1PR axis might be the limited specificity of the S1PR2 antagonist and thus potential adverse effects in non-bone tissues. In this regard, the newly emerging technology of local delivery of S1PRs-targeting drugs via biodegradable polymer scaffolds represents a promising approach for temporal and spatial regulation of bone remodeling [99–101]. As part of this technology, the lipid-based (e.g. FTY720) therapy strategies in *in vivo* models were shown to improve functional recovery of damaged bone by active involvement of the microenvironment through multiple, non-redundant mechanisms including improvements of vascularization and local immune modulation [102–105]. Complementary to the anti-catabolic drug strategy, there is a special interest in novel members of the anabolic class of bone therapeutics. In this regard, S1PR3 could be considered as a candidate target. It has been demonstrated that 8-month-old S1pr3-deficient mice exhibit decreased bone volume and a lower bone formation rate; treatment of wild type but not of S1pr3^−^^/^^−^ osteoblasts with S1P significantly increased mineralization. Importantly, the number and activity of osteoclasts was similar in S1pr3^−^^/^^−^ and wild-type mice. These observations support the notion that selective S1PR3 agonist(s) could be developed as bone anabolic drugs [89].

In this chapter, we reviewed possible applications of our knowledge of the sphingolipid machinery for the design of novel anti-catabolic or anabolic drugs for osteoporosis. Irrespective of the outcome of the future studies that test this approach, we strongly recommend implementing the current evidence interrelating sphingolipid-related mechanisms and bone turnover, into guidelines of clinical trials of sphingolipid-based therapeutics applied in non-bone-related diseases (including autoimmune diseases, chronic inflammatory disorders, and cancer; the therapeutic strategies are reviewed comprehensively in [63,106]). Thus, potential positive or negative effects on bone should be monitored already in early phases of drug development.

As a future perspective, we believe that implementation of systems biology-based approaches through integrative analysis of available data in the ‘omics’ format for further understanding the physiology and pathophysiology of bone, in conjunction with the multifaceted contribution of the sphingolipid machinery will allow to identify novel druggable pathways and targets beyond the prevailing S1P/S1PR axis.

## Article highlights

Imbalances between the activity of osteoblasts and osteoclasts may lead to bone diseases such as osteoporosis, rheumatoid arthritis and bone metastases.

The generation, migration and activity of bone cells is mediated by a variety of molecules including components of the sphingolipid machinery. Recently S1P has been identified as a coupling factor modulating the crosstalk between osteoclasts and osteoblasts.

Since there are numerous unmet needs in the treatment of bone diseases, particularly with regard to osteoporosis, pharmacological interference with the sphingolipid machinery may lead to novel osteotropic therapies.

Summing up cumulative findings from various research areas, our knowledge of S1P/S1PR biology is constantly upgraded; consequently, S1PRs emerge as attractive targets in several diseases.

In context of bone remodeling, implementation of knowledge derived from *in vivo* animal models and *in vitro* studies applying pharmacological agents manipulating the S1P/S1PRs axes allows to consider S1PR2 and S1PR3 as potential drug targets, and, remarkably, by targeting with functional antagonist(s) and agonist(s), respectively.

Given that sphingolipid machinery is druggable at multiple levels, further advances in our understanding of the multifaceted interrelations between the sphingolipid-driven mechanisms and the bone remodeling process might yield novel therapeutic approaches beyond targeting the S1P/S1PR axis in the treatment of bone diseases.

This box summarizes key points contained in the article.

## Glossary

BMD, bone mineral density: BMD is determined by radiologic methods (e.g. dual energy X-ray absorptiometry) and in clinical praxis used as a surrogate for fracture risk.

BMP2, bone morphogenetic protein 2: member of the TGF-β superfamily, osteoinductive, induces the differentiation of osteoblasts.

CXCL12, C-X-C motif chemokine ligand 12, also termed stromal cell-derived growth factor 1 (SDF-1): produced by bone marrow stromal cells; chemotactic factor for lymphocytes and osteoclasts.

Ovariectomy: surgical removal of the ovaries resulting in estrogen deficiency. Since estrogen deficiency is a major pathogenic factor of osteoporosis, ovariectomized rats or mice are standard models of osteoporosis in postmenopausal women.

OPG, osteopotegerin: antagonist of RANKL.

Osteoblast: cell responsible for bone formation. Osteoblasts derive from mesenchymal precursor cells.

Osteoclast: multinucleated bone resorbing cell. Osteoclasts are generated from mononuclear precursor cells in the presence of M-CSF and RANKL.

Osteopetrosis: bone disease characterized by high bone mass.

Osteoporosis: metabolic bone disease characterized by low bone mass and decreased bone strength.

RANKL: factor essential for osteoclast generation.

RANK: receptor for RANKL, expressed on osteoclast precursors.

Vitamin D: a pro-hormone that is metabolized to calcitriol [1α,25(OH)_2_D_3_]. 1α,25(OH)_2_D_3_ is a major calcium-regulating hormone.

S1P, sphingosine 1-phosphate: natural bioactive lipid molecule.

S1PRs, S1P receptors: a family of S1P-specific cell surface G protein-coupled receptors, named S1PR1-5.

SPHK, sphingosine kinase: specific lipid kinase which phosphorylates sphingosine to produce S1P. Two types, SPHK1 and SPHK2, are known.

Sphingolipid machinery: cellular system composed of major lipid mediators within the sphingomyelin/salvage pathway including S1P, the network of sphingolipid-modifying enzymes, S1PRs, and a set of specific transporters.

Sphingolipid-related checkpoints: molecules/mechanisms within the sphingolipid machinery which play regulatory roles in numerous cellular and biological processes including cell proliferation and differentiation, cell survival and apoptosis, cell migration, angiogenesis and immune response/immunomodulation. Among those, S1P/S1PR axes, S1P- and ceramide-producing/modifying enzymes, as well as S1P transporter(s) have been increasingly considered as targets.

LPA, lysophosphatidic acid: bioactive lysophospholipid which shares with S1P a part of enzymatic cellular machinery; binds to specific cell surface G protein-coupled receptors, named LPAR1-6.

FTY720 or [2-amino-2-[2-(4-octylphenyl)ethyl]propane-1,3-diol hydrochloride] (fingolimod, trade name Gilenya, Novartis): pro-drug for the treatment of relapsing forms of multiple sclerosis. FTY720 is a mimetic of natural sphingosine and can be phosphorylated by SPHK2 to produce FTY720-P. FTY720-P acts as a mimetic of S1P and specifically binds to four out of five S1PRs with the exception of S1PR2.

## References

[1] Rauner M, Sipos W, Pietschmann P. Osteoimmunology Int Arch Allergy Immunol. 2007;143(1):31–48. 10.1159/000098223 17191007

[2] Pacifici R. Osteoimmunology and its implications for transplantation. Am J Transplant. 2013 9;13(9):2245–2254. 23915249 10.1111/ajt.12380

[3] Pietschmann P, Mechtcheriakova D, Meshcheryakova A, et al Immunology of Osteoporosis: a Mini-Review. Gerontology. 2016;62(2):128–137. 26088283 10.1159/000431091 PMC4821368

[4] Arron JR, Choi Y. Bone versus immune system. Nature. 2000 11 30;408(6812):535–536. 11117729 10.1038/35046196

[5] Pacifici R T cells: critical bone regulators in health and disease. Bone. 2010 9;47(3):461–471. 20452473 10.1016/j.bone.2010.04.611 PMC2926258

[6] Rauner M, Sipos W, Thiele S, et al Advances in osteoimmunology: pathophysiologic concepts and treatment opportunities. Int Arch Allergy Immunol. 2013;160(2):114–125. 23018236 10.1159/000342426

[7] Sims NA, Martin TJ Coupling the activities of bone formation and resorption: a multitude of signals within the basic multicellular unit. Bonekey Rep. 2014 1;8(3):481. 10.1038/bonekey.2013.215 PMC3899560 24466412

[8] Purdue PE, Crotti TN, Shen Z, et al Comprehensive profiling analysis of actively resorbing osteoclasts identifies critical signaling pathways regulated by bone substrate. Sci Rep. 2014 12;23(4):7595. 10.1038/srep07595 PMC4274512 25534583

[9] Yasuda H, Shima N, Nakagawa N, et al Osteoclast differentiation factor is a ligand for osteoprotegerin/osteoclastogenesis-inhibitory factor and is identical to TRANCE/RANKL. Proc Natl Acad Sci U S A. 1998 3 31;95(7):3597–3602. 9520411 10.1073/pnas.95.7.3597 PMC19881

[10] Martin TJ, Sims NA Osteoclast-derived activity in the coupling of bone formation to resorption. Trends Mol Med. 2005 2;11(2):76–81. 15694870 10.1016/j.molmed.2004.12.004

[11] Odell WD, Heath H 3rd. Osteoporosis: pathophysiology, prevention, diagnosis, and treatment. Disease-A-Month: DM. 1993 11;39(11):789–867. 8223093

[12] Hofbauer LC, Schoppet M Clinical implications of the osteoprotegerin/RANKL/RANK system for bone and vascular diseases. JAMA. 2004 7 28;292(4):490–495. 15280347 10.1001/jama.292.4.490

[13] Rachner TD, Khosla S, Hofbauer LC Osteoporosis: now and the future. Lancet. 2011 4 9; 377(9773):1276–1287. 21450337 10.1016/S0140-6736(10)62349-5 PMC3555696

[14] Sipos W, Pietschmann P, Rauner M, et al Pathophysiology of osteoporosis. Wien Med Wochenschr. 2009 5;159(9–10):230–234. 19484205 10.1007/s10354-009-0647-y

[15] Pietschmann P, Rauner M, Sipos W, et al Osteoporosis: an age-related and gender-specific disease–a mini-review. Gerontology. 2009;55(1):3–12. 18948685 10.1159/000166209

[16] Sobacchi C, Schulz A, Coxon FP, et al Osteopetrosis: genetics, treatment and new insights into osteoclast function. Nat Reviews Endocrinol. 2013 9;9(9):522–536. 10.1038/nrendo.2013.137 23877423

[17] Vallet M, Ralston SH Biology and treatment of paget’s disease of bone. J Cell Biochem. 2016 Feb;117(2):289–299. 26212817 10.1002/jcb.25291

[18] Spiegel S, Milstien S The outs and the ins of sphingosine-1-phosphate in immunity. Nat Reviews Immunol. 2011 6;11(6):403–415. 10.1038/nri2974 PMC3368251 21546914

[19] Blaho VA, Hla T Regulation of mammalian physiology, development, and disease by the sphingosine 1-phosphate and lysophosphatidic acid receptors. Chem Rev. 2011 10 12;111(10):6299–6320. 21939239 10.1021/cr200273u PMC3216694

[20] Hla T, Maciag T An abundant transcript induced in differentiating human endothelial cells encodes a polypeptide with structural similarities to G-protein-coupled receptors. J Biol Chem. 1990 6 5;265(16):9308–9313. 2160972

[21] Lee MJ, Van Brocklyn JR, Thangada S, et al Sphingosine-1-phosphate as a ligand for the G protein-coupled receptor EDG-1. Science. 1998 3 6;279(5356):1552–1555. 9488656 10.1126/science.279.5356.1552

[22] Strub GM, Maceyka M, Hait NC, et al Extracellular and intracellular actions of sphingosine-1-phosphate. Adv Exp Med Biol. 2010;688:141–155. 20919652 10.1007/978-1-4419-6741-1_10 PMC2951632

[23] Spiegel S, Milstien S Functions of the multifaceted family of sphingosine kinases and some close relatives. J Biol Chem. 2007 1 26;282(4):2125–2129. 17135245 10.1074/jbc.R600028200

[24] Van Veldhoven PP, Mannaerts GP Sphingosine-phosphate lyase. Adv Lipid Res. 1993;26:69–98. 8379460

[25] Zhou J, Saba JD Identification of the first mammalian sphingosine phosphate lyase gene and its functional expression in yeast. Biochem Biophys Res Commun. 1998 1 26;242(3):502–507. 9464245 10.1006/bbrc.1997.7993

[26] Ogawa C, Kihara A, Gokoh M, et al Identification and characterization of a novel human sphingosine-1-phosphate phosphohydrolase, hSPP2. J Biol Chem. 2003 1 10;278(2):1268–1272. 12411432 10.1074/jbc.M209514200

[27] Mandala SM, Thornton R, Galve-Roperh I, et al Molecular cloning and characterization of a lipid phosphohydrolase that degrades sphingosine-1- phosphate and induces cell death. Proc Natl Acad Sci U S A. 2000 7 5;97(14):7859–7864. 10859351 10.1073/pnas.120146897 PMC16635

[28] Mechtcheriakova D, Wlachos A, Sobanov J, et al Sphingosine 1-phosphate phosphatase 2 is induced during inflammatory responses. Cell Signal. 2007 4;19(4):748–760. 17113265 10.1016/j.cellsig.2006.09.004

[29] Le Stunff H, Peterson C, Liu H, et al Sphingosine-1-phosphate and lipid phosphohydrolases. Biochim Biophys Acta. 2002 5 23;1582(1–3):8–17. 12069805 10.1016/s1388-1981(02)00132-4

[30] Brindley DN, Pilquil C Lipid phosphate phosphatases and signaling. J Lipid Res. 2009 4;50(Suppl):S225–30. 19066402 10.1194/jlr.R800055-JLR200 PMC2674702

[31] Pappu R, Schwab SR, Cornelissen I, et al Promotion of lymphocyte egress into blood and lymph by distinct sources of sphingosine-1-phosphate. Science. 2007 4 13;316(5822):295–298. 17363629 10.1126/science.1139221

[32] Urtz N, Gaertner F, Von Bruehl ML, et al Sphingosine 1-phosphate produced by sphingosine kinase 2 intrinsically controls platelet aggregation in vitro and in vivo. Circ Res. 2015 7 31;117(4):376–387. 26129975 10.1161/CIRCRESAHA.115.306901

[33] Dimitriou R, Tsiridis E, Giannoudis PV Current concepts of molecular aspects of bone healing. Injury. 2005 12;36(12):1392–1404. 16102764 10.1016/j.injury.2005.07.019

[34] Ryu J, Kim HJ, Chang EJ, et al Sphingosine 1-phosphate as a regulator of osteoclast differentiation and osteoclast-osteoblast coupling. Embo J. 2006 12 13; 25(24):5840–5851. 17124500 10.1038/sj.emboj.7601430 PMC1698879

[35] Brizuela L, Martin C, Jeannot P, et al Osteoblast-derived sphingosine 1-phosphate to induce proliferation and confer resistance to therapeutics to bone metastasis-derived prostate cancer cells. Mol Oncol. 2014 10;8(7):1181–1195. 24768038 10.1016/j.molonc.2014.04.001 PMC5528572

[36] Schwab SR, Cyster JG Finding a way out: lymphocyte egress from lymphoid organs. Nat Immunol. 2007 12;8(12):1295–1301. 18026082 10.1038/ni1545

[37] Albinet V, Bats ML, Huwiler A, et al Dual role of sphingosine kinase-1 in promoting the differentiation of dermal fibroblasts and the dissemination of melanoma cells. Oncogene. 2014 6 26;33(26):3364–3373. 23893239 10.1038/onc.2013.303

[38] Meshcheryakova A, Svoboda M, Tahir A, et al Exploring the role of sphingolipid machinery during the epithelial to mesenchymal transition program using an integrative approach. Oncotarget. 2016 4 19;7(16):22295–22323. 26967245 10.18632/oncotarget.7947 PMC5008362

[39] Brinkmann V, Billich A, Baumruker T, et al Fingolimod (FTY720): discovery and development of an oral drug to treat multiple sclerosis. Nat Rev Drug Discov. 2010 11;9(11):883–897. 21031003 10.1038/nrd3248

[40] Zemann B, Kinzel B, Muller M, et al Sphingosine kinase type 2 is essential for lymphopenia induced by the immunomodulatory drug FTY720. Blood. 2006 2 15;107(4):1454–1458. 16223773 10.1182/blood-2005-07-2628

[41] Mechtcheriakova D, Wlachos A, Sobanov J, et al FTY720-phosphate is dephosphorylated by lipid phosphate phosphatase 3. FEBS Lett. 2007 6 26;581(16):3063–3068. 17555747 10.1016/j.febslet.2007.05.069

[42] Brinkmann V, Davis MD, Heise CE, et al The immune modulator FTY720 targets sphingosine 1-phosphate receptors. J Biol Chem. 2002 6 14;277(24):21453–21457. 11967257 10.1074/jbc.C200176200

[43] Oo ML, Thangada S, Wu MT, et al Immunosuppressive and anti-angiogenic sphingosine 1-phosphate receptor-1 agonists induce ubiquitinylation and proteasomal degradation of the receptor. J Biol Chem. 2007 3 23;282(12):9082–9089. 17237497 10.1074/jbc.M610318200

[44] Brinkmann V, Cyster JG, Hla T FTY720: sphingosine 1-phosphate receptor-1 in the control of lymphocyte egress and endothelial barrier function. Am J Transplant. 2004 7;4(7):1019–1025. 15196057 10.1111/j.1600-6143.2004.00476.x

[45] Jo E, Sanna MG, Gonzalez-Cabrera PJ, et al S1P1-selective in vivo-active agonists from high-throughput screening: off-the-shelf chemical probes of receptor interactions, signaling, and fate. Chem Biol. 2005 6;12(6):703–715. 15975516 10.1016/j.chembiol.2005.04.019

[46] Thangada S, Khanna KM, Blaho VA, et al Cell-surface residence of sphingosine 1-phosphate receptor 1 on lymphocytes determines lymphocyte egress kinetics. J Exp Med. 2010 7 5;207(7):1475–1483. 20584883 10.1084/jem.20091343 PMC2901064

[47] Mandala S, Hajdu R, Bergstrom J, et al Alteration of lymphocyte trafficking by sphingosine-1-phosphate receptor agonists. Science. 2002 4 12;296(5566):346–349. 11923495 10.1126/science.1070238

[48] White C, Alshaker H, Cooper C, et al The emerging role of FTY720 (Fingolimod) in cancer treatment. Oncotarget. 2016 4 26;7(17):23106–23127. 27036015 10.18632/oncotarget.7145 PMC5029614

[49] Pchejetski D, Bohler T, Brizuela L, et al FTY720 (fingolimod) sensitizes prostate cancer cells to radiotherapy by inhibition of sphingosine kinase-1. Cancer Res. 2010 11 1;70(21):8651–8661. 20959468 10.1158/0008-5472.CAN-10-1388

[50] Xia P, Wang L, Moretti PA, et al Sphingosine kinase interacts with TRAF2 and dissects tumor necrosis factor-alpha signaling. J Biol Chem. 2002 3 8;277(10):7996–8003. 11777919 10.1074/jbc.M111423200

[51] Alvarez SE, Harikumar KB, Hait NC, et al Sphingosine-1-phosphate is a missing cofactor for the E3 ubiquitin ligase TRAF2. Nature. 2010 6 24;465(7301):1084–1088. 20577214 10.1038/nature09128 PMC2946785

[52] Yu X, Huang Y, Collin-Osdoby P, et al Stromal cell-derived factor-1 (SDF-1) recruits osteoclast precursors by inducing chemotaxis, matrix metalloproteinase-9 (MMP-9) activity, and collagen transmigration. J Bone Miner Res. 2003 8;18(8):1404–1418. 12929930 10.1359/jbmr.2003.18.8.1404

[53] Wright LM, Maloney W, Yu X, et al Stromal cell-derived factor-1 binding to its chemokine receptor CXCR4 on precursor cells promotes the chemotactic recruitment, development and survival of human osteoclasts. Bone. 2005 5;36(5):840–853. 15794931 10.1016/j.bone.2005.01.021

[54] Lean JM, Murphy C, Fuller K, et al CCL9/MIP-1gamma and its receptor CCR1 are the major chemokine ligand/receptor species expressed by osteoclasts. J Cell Biochem. 2002;87(4):386–393. 12397598 10.1002/jcb.10319

[55] Blaho VA, Hla T An update on the biology of sphingosine 1-phosphate receptors. J Lipid Res. 2014 1 23; 55(8):1596–1608. 24459205 10.1194/jlr.R046300 PMC4109755

[56] Chi H Sphingosine-1-phosphate and immune regulation: trafficking and beyond. Trends Pharmacol Sci. 2011 1;32(1):16–24. 21159389 10.1016/j.tips.2010.11.002 PMC3017656

[57] Ishii M, Kikuta J, Shimazu Y, et al Chemorepulsion by blood S1P regulates osteoclast precursor mobilization and bone remodeling in vivo. J Exp Med. 2010 12 20; 207(13):2793–2798. 21135136 10.1084/jem.20101474 PMC3005230

[58] Ishii M, Egen JG, Klauschen F, et al Sphingosine-1-phosphate mobilizes osteoclast precursors and regulates bone homeostasis. Nature. 2009 3 26;458(7237):524–528. 19204730 10.1038/nature07713 PMC2785034

[59] Wei SH, Rosen H, Matheu MP, et al Sphingosine 1-phosphate type 1 receptor agonism inhibits transendothelial migration of medullary T cells to lymphatic sinuses. Nat Immunol. 2005 12;6(12):1228–1235. 16273098 10.1038/ni1269

[60] Jung S, Aliberti J, Graemmel P, et al Analysis of fractalkine receptor CX(3)CR1 function by targeted deletion and green fluorescent protein reporter gene insertion. Mol Cell Biol. 2000 6;20(11):4106–4114. 10805752 10.1128/mcb.20.11.4106-4114.2000 PMC85780

[61] Burnett SH, Kershen EJ, Zhang J, et al Conditional macrophage ablation in transgenic mice expressing a Fas-based suicide gene. J Leukoc Biol. 2004 4;75(4):612–623. 14726498 10.1189/jlb.0903442

[62] Schwab SR, Pereira JP, Matloubian M, et al Lymphocyte sequestration through S1P lyase inhibition and disruption of S1P gradients. Science. 2005 Sep 9;309(5741):1735–1739. 16151014 10.1126/science.1113640

[63] Kunkel GT, Maceyka M, Milstien S, et al Targeting the sphingosine-1-phosphate axis in cancer, inflammation and beyond. Nat Rev Drug Discov. 2013 9;12(9):688–702. 23954895 10.1038/nrd4099 PMC3908769

[64] Osada M, Yatomi Y, Ohmori T, et al Enhancement of sphingosine 1-phosphate-induced migration of vascular endothelial cells and smooth muscle cells by an EDG-5 antagonist. Biochem Biophys Res Commun. 2002 12 6;299(3):483–487. 12445827 10.1016/s0006-291x(02)02671-2

[65] Ishii T, Shimazu Y, Nishiyama I, et al The role of sphingosine 1-phosphate in migration of osteoclast precursors; an application of intravital two-photon microscopy. Mol Cells. 2011 5;31(5):399–403. 21360199 10.1007/s10059-011-1010-x PMC3887611

[66] Kono M, Mi Y, Liu Y, et al The sphingosine-1-phosphate receptors S1P1, S1P2, and S1P3 function coordinately during embryonic angiogenesis. J Biol Chem. 2004 7 9;279(28):29367–29373. 15138255 10.1074/jbc.M403937200

[67] Tomimori Y, Mori K, Koide M, et al Evaluation of pharmaceuticals with a novel 50-hour animal model of bone loss. J Bone Miner Res. 2009 7;24(7):1194–1205. 19257825 10.1359/jbmr.090217

[68] Maeda Y, Seki N, Sato N, et al Sphingosine 1-phosphate receptor type 1 regulates egress of mature T cells from mouse bone marrow. Int Immunol. 2010 6;22(6):515–525. 20497959 10.1093/intimm/dxq036

[69] Travlos GS Normal structure, function, and histology of the bone marrow. Toxicol Pathol. 2006;34(5):548–565. 17067943 10.1080/01926230600939856

[70] Plum LA, DeLuca HF, Vitamin D Disease and therapeutic opportunities. Nat Rev Drug Discov. 2010 12;9(12):941–955. 21119732 10.1038/nrd3318

[71] Kikuta J, Kawamura S, Okiji F, et al Sphingosine-1-phosphate-mediated osteoclast precursor monocyte migration is a critical point of control in antibone-resorptive action of active vitamin D. Proc Natl Acad Sci U S A. 2013 4 23;110(17):7009–7013. 23569273 10.1073/pnas.1218799110 PMC3637769

[72] Takasu H, Sugita A, Uchiyama Y, et al c-Fos protein as a target of anti-osteoclastogenic action of vitamin D, and synthesis of new analogs. J Clin Invest. 2006 2;116(2):528–535. 16424941 10.1172/JCI24742 PMC1332025

[73] Harada S, Mizoguchi T, Kobayashi Y, et al Daily administration of eldecalcitol (ED-71), an active vitamin D analog, increases bone mineral density by suppressing RANKL expression in mouse trabecular bone. J Bone Miner Res. 2012 2;27(2):461–473. 22052469 10.1002/jbmr.555

[74] Teti A Mechanisms of osteoclast-dependent bone formation. Bonekey Rep. 2013;2:449. 24422142 10.1038/bonekey.2013.183 PMC3872977

[75] Pederson L, Ruan M, Westendorf JJ, et al Regulation of bone formation by osteoclasts involves Wnt/BMP signaling and the chemokine sphingosine-1-phosphate. Proc Natl Acad Sci U S A. 2008 12 30;105(52):20764–20769. 19075223 10.1073/pnas.0805133106 PMC2603259

[76] Quint P, Ruan M, Pederson L, et al Sphingosine 1-phosphate (S1P) receptors 1 and 2 coordinately induce mesenchymal cell migration through S1P activation of complementary kinase pathways. J Biol Chem. 2013 2 22;288(8):5398–5406. 23300082 10.1074/jbc.M112.413583 PMC3581421

[77] Vaes BL, Dechering KJ, Feijen A, et al Comprehensive microarray analysis of bone morphogenetic protein 2-induced osteoblast differentiation resulting in the identification of novel markers for bone development. J Bone Miner Res. 2002 12;17(12):2106–2118. 12469905 10.1359/jbmr.2002.17.12.2106

[78] Roelofsen T, Akkers R, Beumer W, et al Sphingosine-1-phosphate acts as a developmental stage specific inhibitor of platelet-derived growth factor-induced chemotaxis of osteoblasts. J Cell Biochem. 2008 11 1;105(4):1128–1138. 18819098 10.1002/jcb.21915

[79] Ohmori T, Yatomi Y, Osada M, et al Sphingosine 1-phosphate induces contraction of coronary artery smooth muscle cells via S1P2. Cardiovasc Res. 2003 4 1;58(1):170–177. 12667959 10.1016/s0008-6363(03)00260-8

[80] Grey A, Xu X, Hill B, et al Osteoblastic cells express phospholipid receptors and phosphatases and proliferate in response to sphingosine-1-phosphate. Calcif Tissue Int. 2004 6;74(6):542–550. 15354862 10.1007/s00223-003-0155-9

[81] Grey A, Banovic T, Naot D, et al Lysophosphatidic acid is an osteoblast mitogen whose proliferative actions involve G(i) proteins and protein kinase C, but not P42/44 mitogen-activated protein kinases. Endocrinology. 2001 3;142(3):1098–1106. 11181524 10.1210/endo.142.3.8011

[82] Lampasso JD, Kamer A, Margarone J 3rd, et al Sphingosine-1-phosphate effects on PKC isoform expression in human osteoblastic cells. Prostaglandins Leukot Essent Fatty Acids. 2001 9;65(3):139–146. 11728164 10.1054/plef.2001.0302

[83] Lampasso JD, Marzec N, Margarone J 3rd, et al Role of protein kinase C alpha in primary human osteoblast proliferation. J Bone Miner Res. 2002 11;17(11):1968–1976. 12412804 10.1359/jbmr.2002.17.11.1968

[84] Manolagas SC Birth and death of bone cells: basic regulatory mechanisms and implications for the pathogenesis and treatment of osteoporosis. Endocr Rev. 2000 4;21(2):115–137. 10782361 10.1210/edrv.21.2.0395

[85] Grey A, Chen Q, Callon K, et al The phospholipids sphingosine-1-phosphate and lysophosphatidic acid prevent apoptosis in osteoblastic cells via a signaling pathway involving G(i) proteins and phosphatidylinositol-3 kinase. Endocrinology. 2002 12;143(12):4755–4763. 12446603 10.1210/en.2002-220347

[86] Salles JP, Laurencin-Dalicieux S, Conte-Auriol F, et al Bone defects in LPA receptor genetically modified mice. Biochim Biophys Acta. 2013 1;1831(1):93–98. 22867754 10.1016/j.bbalip.2012.07.018

[87] Sims SM, Panupinthu N, Lapierre DM, et al Lysophosphatidic acid: a potential mediator of osteoblast-osteoclast signaling in bone. Biochim Biophys Acta. 2013 1;1831(1):109–116. 22892679 10.1016/j.bbalip.2012.08.001

[88] Lotinun S, Kiviranta R, Matsubara T, et al Osteoclast-specific cathepsin K deletion stimulates S1P-dependent bone formation. J Clin Invest. 2013 2;123(2):666–681. 23321671 10.1172/JCI64840 PMC3561821

[89] Keller J, Catala-Lehnen P, Huebner AK, et al Calcitonin controls bone formation by inhibiting the release of sphingosine 1-phosphate from osteoclasts. Nat Commun. 2014;5:5215. 25333900 10.1038/ncomms6215 PMC4205484

[90] Fuller K, Lawrence KM, Ross JL, et al Cathepsin K inhibitors prevent matrix-derived growth factor degradation by human osteoclasts. Bone. 2008 1;42(1):200–211. 17962093 10.1016/j.bone.2007.09.044

[91] Marx SJ, Woodward CJ, Aurbach GD Calcitonin receptors of kidney and bone. Science. 1972 12 1;178(4064):999–1001. 5084670 10.1126/science.178.4064.999

[92] Wuster C, Raue F, Meyer C, et al Long-term excess of endogenous calcitonin in patients with medullary thyroid carcinoma does not affect bone mineral density. J Endocrinol. 1992 7;134(1):141–147. 1354241 10.1677/joe.0.1340141

[93] Lee SH, Lee SY, Lee YS, et al Higher circulating sphingosine 1-phosphate levels are associated with lower bone mineral density and higher bone resorption marker in humans. J Clin Endocrinol Metab. 2012 8;97(8):E1421–8. 22679064 10.1210/jc.2012-1044

[94] Kim BJ, Koh JM, Lee SY, et al Plasma sphingosine 1-phosphate levels and the risk of vertebral fracture in postmenopausal women. J Clin Endocrinol Metab. 2012 10;97(10):3807–3814. 22879631 10.1210/jc.2012-2346

[95] Kanis JA, McCloskey EV, Johansson H, et al European guidance for the diagnosis and management of osteoporosis in postmenopausal women. Osteoporos Int. 2013 1;24(1):23–57. 23079689 10.1007/s00198-012-2074-y PMC3587294

[96] Koberlin MS, Snijder B, Heinz LX, et al A conserved circular network of coregulated lipids modulates innate immune responses. Cell. 2015 7 2;162(1):170–183. 26095250 10.1016/j.cell.2015.05.051 PMC4523684

[97] Hernlund E, Svedbom A, Ivergard M, et al Osteoporosis in the European Union: medical management, epidemiology and economic burden. A report prepared in collaboration with the International Osteoporosis Foundation (IOF) and the European Federation of Pharmaceutical Industry Associations (EFPIA). Archives of osteoporosis 2013;8:136. 24113837 10.1007/s11657-013-0136-1 PMC3880487

[98] Riggs BL, Parfitt AM Drugs used to treat osteoporosis: the critical need for a uniform nomenclature based on their action on bone remodeling. J Bone Miner Res. 2005 2;20(2):177–184. 15647810 10.1359/JBMR.041114

[99] Gentile P, Chiono V, Carmagnola I, et al An overview of poly(lactic-co-glycolic) acid (PLGA)-based biomaterials for bone tissue engineering. Int J Mol Sci. 2014 2 28;15(3):3640–3659. 24590126 10.3390/ijms15033640 PMC3975359

[100] Das A, Tanner S, Barker DA, et al Delivery of S1P receptor-targeted drugs via biodegradable polymer scaffolds enhances bone regeneration in a critical size cranial defect. J Biomed Mater Res A. 2014 4;102(4):1210–1218. 23640833 10.1002/jbm.a.34779 PMC3951302

[101] Huang C, Das A, Barker D, et al Local delivery of FTY720 accelerates cranial allograft incorporation and bone formation. Cell Tissue Res. 2012 3;347(3):553–566. 21863314 10.1007/s00441-011-1217-3 PMC3464023

[102] Das A, Segar CE, Hughley BB, et al The promotion of mandibular defect healing by the targeting of S1P receptors and the recruitment of alternatively activated macrophages. Biomaterials. 2013 12;34(38):9853–9862. 24064148 10.1016/j.biomaterials.2013.08.015 PMC3797185

[103] Das A, Segar CE, Chu Y, et al Bioactive lipid coating of bone allografts directs engraftment and fate determination of bone marrow-derived cells in rat GFP chimeras. Biomaterials. 2015;64:98–107. 26125501 10.1016/j.biomaterials.2015.06.019 PMC4551493

[104] Das A, Barker DA, Wang T, et al Delivery of bioactive lipids from composite microgel-microsphere injectable scaffolds enhances stem cell recruitment and skeletal repair. PLoS One. 2014;9(7):e101276. 25077607 10.1371/journal.pone.0101276 PMC4117484

[105] Wang T, Krieger J, Huang C, et al Enhanced osseous integration of human trabecular allografts following surface modification with bioactive lipids. Drug Deliv Transl Res. 2016 4;6(2):96–104. 26169381 10.1007/s13346-015-0244-0 PMC5472849

[106] Bigaud M, Guerini D, Billich A, et al Second generation S1P pathway modulators: research strategies and clinical developments. Biochim Biophys Acta. 2014 5;1841(5):745–758. 24239768 10.1016/j.bbalip.2013.11.001

